# Delayed Withdrawal of Life-Sustaining Treatment in Disorders of Consciousness: Practical and Theoretical Considerations

**DOI:** 10.1007/s12028-024-02143-7

**Published:** 2024-10-15

**Authors:** Aaron Williams, Geoffrey D. Bass, Stephen Hampton, Rachel Klinedinst, Joseph T. Giacino, David Fischer

**Affiliations:** 1 Department of Neurology, Perelman School of Medicine at the University of Pennsylvania, Philadelphia, PA USA; 2 Division of Pulmonary, Allergy, and Critical Care, Department of Medicine, Perelman School of Medicine at the University of Pennsylvania, Philadelphia, PA USA; 3 Department of Physical Medicine and Rehabilitation, Perelman School of Medicine at the University of Pennsylvania, Philadelphia, PA USA; 4 Division of Palliative Care, Department of Medicine, Hospital of the University of Pennsylvania, Philadelphia, PA USA; 5 Department of Physical Medicine and Rehabilitation, Spaulding Rehabilitation Hospital, Massachusetts General Hospital, Harvard Medical School, Boston, MA USA

**Keywords:** Acute brain injury, Disorder of consciousness, Prognostic factors, Life support care, Withdrawing care

## Abstract

Disorders of consciousness (DoC) resulting from severe acute brain injuries may prompt clinicians and surrogate decision makers to consider withdrawal of life-sustaining treatment (WLST) if the neurologic prognosis is poor. Recent guidelines suggest, however, that clinicians should avoid definitively concluding a poor prognosis prior to 28 days post injury, as patients may demonstrate neurologic recovery outside the acute time period. This practice may increase the frequency with which clinicians consider the option of delayed WLST (D-WLST), namely, WLST that would occur after hospital discharge, if the patient’s recovery trajectory ultimately proves inconsistent with an acceptable quality of life. However acute care clinicians are often uncertain about what D-WLST entails and therefore find it difficult to properly counsel surrogates about this option. Here, we describe practical and theoretical considerations relevant to D-WLST. We first identify post-acute-care facilities to which patients with DoC are likely to be discharged and where D-WLST may be considered. Second, we describe how clinicians and surrogates may determine the appropriate timing of D-WLST. Third, we outline how D-WLST is practically implemented. And finally, we discuss psychosocial barriers to D-WLST, including the regret paradox, in which surrogates of patients who do not recover to meet preestablished goals frequently choose not to ultimately pursue D-WLST. Together, these practical, logistic, and psychosocial factors must be considered when potentially deferring WLST to the post-acute-care setting to optimize neurologic recovery for patients, avoid prolonged undue suffering, and promote informed and shared decision-making between clinicians and surrogates.

## Introduction

Severe acute brain injuries (SABI) [1], which include hypoxic-ischemic brain injury (HIBI), traumatic brain injury (TBI), intracranial hemorrhage, and stroke, often result in disorders of consciousness (DoC). For patients with SABI and DoC and a poor neurologic prognosis, clinicians and surrogate decision makers (surrogates) may consider withdrawal of life-sustaining treatment (WLST). The timing of this decision is critical. Guidelines have historically recommended deferring WLST for at least 72 h after SABI [1] to allow the completion of acute therapies and collection of prognostic data [2]. The majority of WLST decisions have been made shortly after this time period, within a week of the SABI [3, 4]. However, recognizing that recovery for many patients occurs after this period [5, 6], more recent guidelines from the American Academy of Neurology, the American Congress of Rehabilitation Medicine, and the National Institute on Disability, Independent Living, and Rehabilitation Research suggest waiting at least 28 days before definitively presuming a poor prognosis and offering WLST [7]. One approach to accommodating this recommendation is a time-limited trial in which life-sustaining treatment (LST) is continued for a prespecified period of time, after which goal attainment is reassessed to determine the appropriateness of WLST [8]. Even in cases in which early clinical evidence strongly suggests a poor prognosis, surrogates may need more time to emotionally process the situation or gather additional information before pursuing WLST.

Consequently, decisions about WLST for patients with DoC may be postponed until after acute care hospital discharge. However, for most surrogates and acute care clinicians, delayed WLST (D-WLST)—that is, WLST that occurs after hospital discharge—and the process by which it is considered and implemented is unknown. Surrogates therefore do not know what to expect, and clinicians may have difficulty providing counsel, potentially causing surrogates distress and reducing adherence to clinical guidelines. A clearer understanding of what D-WLST entails may help surrogates and clinicians make more informed decisions about this option. Here, we discuss the practical and theoretical considerations relevant to D-WLST by outlining the types of post-acute-care facilities where D-WLST may be considered, discussing the logistical and psychosocial elements of D-WLST, and suggesting future directions for clinical care and research. We focus on health care facilities and logistic considerations relevant to the United States health care system, though many of the principles may apply to clinical situations abroad as well. Our aim is to provide acute care clinicians with a road map for navigating these complex decisions surrounding D-WLST and the tools for counseling surrogates accordingly.

## Settings Where D-WLST May Occur

### Inpatient Rehabilitation Facilities

**Fig. 1:**
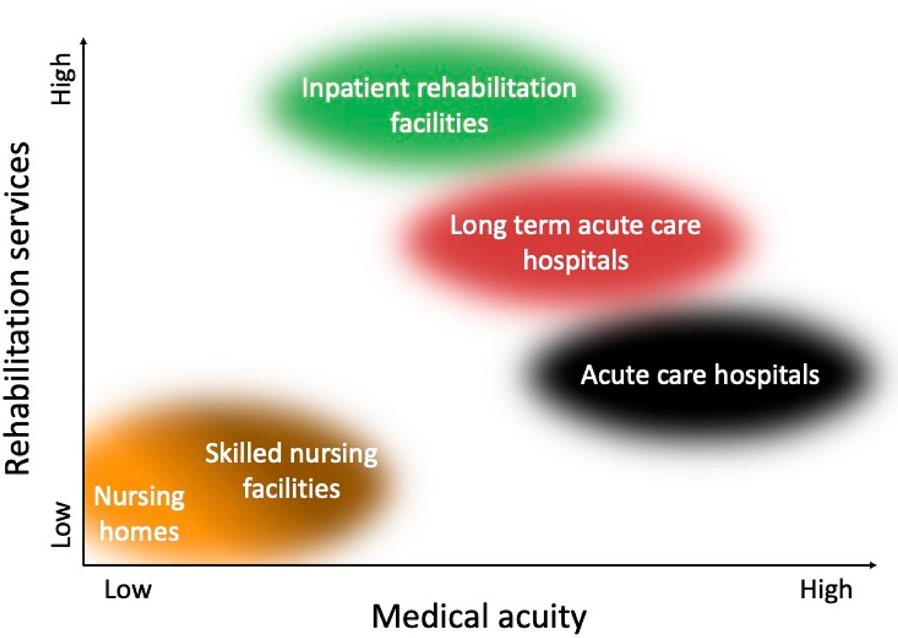
Medical and rehabilitative services vary across post-acute-care facilities. Acute care hospitals are capable of providing the highest acuity medical services, with long-term acute care hospitals often serving as a discharge destination still capable of complex medical management and rehabilitation. Inpatient rehabilitation facilities primarily function as rehabilitative facilities for transitions toward functional autonomy. Skilled nursing facilities vary by region and policies but largely care for medically stable patients without intensive rehabilitative services and are often co-housed in or are dually certified as long-term care facilities (i.e., nursing homes). Some long-term acute care and inpatient rehabilitation facilities offer disorders of consciousness programs, which provide rehabilitative services specific to patients with severe neurologic injury

**Fig. 2:**
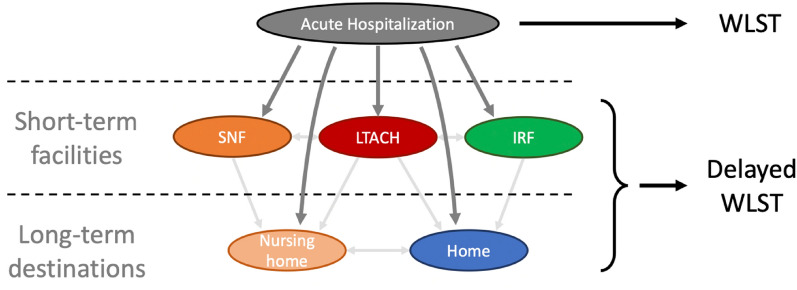
Post-acute-care facilities of disorders of consciousness (DoC) recovery. After sustaining severe acute brain injury and DoC, patients may be discharged to a post-acute-care facility or home, each with a typical patient population and each capable of specific types of medical and rehabilitative services. Patients often move between multiple facilities after the hospitalization (only a portion of possible transfers are represented by arrows). Delayed withdrawal of life-sustaining treatment (WLST), defined as WLST that occurs after the acute care hospitalization, will differ in how it is considered and executed across these different sites of care. *IRF* inpatient rehabilitation facility, *LTACH* long-term acute care hospital, *SNF* skilled nursing facility

Inpatient rehabilitation facilities (IRFs), also known as acute care rehabilitation hospitals, provide intensive short-term rehabilitation services for patients with a range of medical conditions [9] (Table 1, Fig. 1,  and Fig. 2). Though recoveries of patients admitted to IRF with neurologic injuries vary widely, studies describe trends of those trajectories [10]. Demographic and clinical factors, such as younger age, fewer comorbidities, male sex, and shorter acute care hospitalization, are predictive of discharge to IRF both for patients with moderate to severe TBI and for those with HIBI [10–12]. Once at an IRF, rehabilitation seems to similarly benefit those with severe TBI and HIBI in terms of recovery of functional independence [13, 14]. Early rehabilitation for stroke also has similarly demonstrated benefits, though the extent to which these benefits extend to DoC in particular is less well described [15]. Notably, discharge trends both to and from IRFs may be driven not only by clinical factors, such as recovery, but also by factors such as insurance coverage, accessible resources, geographic factors, and bed availability.

**Table 1:** Post-acute-care facilities

Facility	Description	Typical goals	Typical clinicians
IRF	Short-term rehabilitation, typically for patients able to participate in intensive rehab (often 3 h per day, 5 days per week)	Intensive physical, occupational, and speech therapy	Physiatrists, involved regularly
LTACH	Short-term medical care for patients discharged from an acute care hospital with ongoing complex medical needs	Mechanical ventilation management and weaning Stabilization of complex medical needs Management of chronic organ failure	Hospitalists, involved regularly
SNF	Short-term medical and nursing care for simple, stable conditions; some SNFs are dually certified as long-term care facilities (i.e., nursing homes)	Management of stable wounds Cleaning assistance, bed transfer	Physician (e.g., geriatrician, internist), involved infrequently
DoC programs	Short-term specialized medical and rehabilitative services for patients with DoC; often a component of IRF or LTACH	Specialized physical, occupational, and speech therapy for DoC Detailed assessments to detect and promote recovery of consciousness	Clinician (e.g., physiatrist, neurologist, neuropsychologist) with expertise in DoC, involved regularly

*DoC* disorders of consciousness, *IRF* inpatient rehabilitation facility, *LTACH* long-term acute care hospital, *SNF* skilled nursing facility

The recovery of consciousness is often a pivotal factor in whether families consider D-WLST. In a study of more than 17,000 patients with moderate to severe TBI, 12% had a DoC at the time of transfer to an IRF [16]. Of these patients, 82% recovered consciousness while at the IRF. Even for patients with TBI who did not follow commands at an IRF, a multisite cohort study demonstrated that by 10 years post injury, one third were independently dressing and more than half were independently toileting [17]. Nontraumatic causes of DoC, including HIBI and stroke, have lower rates of consciousness recovery [18, 19]. It remains uncertain if IRF admission per se affects rates of consciousness recovery across etiologies, a topic that warrants further investigation.

There are limited data on D-WLST decisions in the IRF setting, potentially because IRFs are designed for restoration of function and therefore may be less likely to consider D-WLST. Factors influencing decisions about D-WLST, such as current neurologic status and recovery trajectory, may be readily assessed at IRF programs. Physiatrists, especially at IRFs that receive patients with neurologic injuries, are familiar with the management, sequelae, and challenges of neurologic recovery. However, comfort with mediating discussions about D-WLST may be less consistent. Other factors potentially relevant to D-WLST decisions, such as systemic medical complications, may be less readily assessable and manageable at an IRF. Whether and how D-WLST is considered in IRFs warrants further investigation.

### Long-Term Acute Care Hospitals

Long-term acute care hospitals (LTACHs) manage patients who are discharged from acute care hospitals but continue to require complex inpatient medical care [20] (Table 1, Fig. 1, and Fig. 2). Data on mortality are more widely available for LTACHs than for IRFs. Patients admitted to LTACHs generally have a high mortality rate, ranging from 40% 90-day mortality among mechanically ventilated patients [21] to 69%, 66%, and 63% 1-year mortality among patients with a tracheostomy, gastrotomy tube, or both [20]. Another study found that the median survival for patients over the ages of 65 admitted to an LTACH was 8.3 months, with 1- and 5-year survival being 45% and 18%, respectively [22]. Patients initially admitted to an LTACH often express goals of speaking, eating/drinking, walking, self-toileting, and home discharge; by LTACH discharge, achievement of these goals was 97%, 88%, 21%, 18%, and 13%, respectively, in a single-center report [23]. Liberation from mechanical ventilation while in an LTACH is estimated at 51.7% [21]. These statistics highlight the importance of setting realistic expectations and revisiting progress toward goal attainment and quality of life for patients admitted to LTACHs. LTACHs are often staffed by hospitalists who may assist in discussions of D-WLST, as necessary.

An important caveat to these figures is that most studies on patient outcomes from LTACHs include patients with a wide range of medical conditions not limited to neurologic injury or DoC, and some studies specifically exclude patients who cannot communicate. Therefore, the functional outcome and mortality data specific to patients with DoC may differ. Indeed, LTACH length of stay is longer and mortality rate is higher for patients with more severe cognitive dysfunction [24]. Additionally, many LTACH studies are conducted in more elderly populations [20, 22], which may confound these outcomes. Estimates and predictors of continued neurologic recovery warrant further investigation to help inform decisions about D-WLST in this setting.

### Skilled Nursing Facilities and Long-Term Care Facilities

Skilled nursing facilities (SNFs) support a range of medical and nursing needs following hospitalization that are less complex than those at LTACHs [9] (Table 1, Fig. 1, and Fig. 2). Several factors influence whether patients are discharged to a SNF and the outcomes of those patients. A higher number and severity of comorbid conditions increases the chance of admission to a SNF compared to an IRF following acute care hospital discharge [25]. In a study of 285 patients with severe TBI, nonneurologic organ dysfunction during the intensive care unit (ICU) stay resulted in a higher likelihood of discharge to a SNF compared to those without nonneurologic organ dysfunction [26]. For Medicare patients with TBI, factors such as incontinence, decreased functional independence, and impaired cognition were negatively associated with successful community discharge from a SNF without death or readmission [27]. Nonclinical factors, such as insurance and payer authorization, also play large roles in determining patient disposition following acute care hospitalization [28]. Although less intensive than that provided at IRFs or LTACHs, the rehabilitation provided at SNFs may be impactful; in a retrospective study of more than 23,000 patients with stroke, early and regular rehabilitation was associated with an increased likelihood of being discharged home from a SNF [29].

There are limited data specific to mortality and D-WLST for patients with DoC in SNFs. Of all patients admitted to an SNF from an ICU with a tracheostomy, gastrostomy, or both, 1-year mortality was 44%, 52%, and 56%, respectively [20]. These data are slightly more optimistic than those for patients admitted to LTACHs, with a similar caveat that trends for DoC specifically may differ. Nevertheless, they indicate that morbidity and mortality remain high in this setting, highlighting the importance of setting realistic expectations for surrogates. Importantly, in contrast to IRFs and LTACHs, clinician oversight at SNFs is less intensive. According to the Center for Medicare and Medicaid Services, visits by physicians or associated medical providers at SNFs and long-term care facilities are only required every 30 days, which may hamper consideration and discussions of D-WLST when appropriate.

### DoC Programs

DoC programs offer specialized medical and rehabilitative services for patients with DoC, typically as part of an IRF or LTACH. DoC includes coma, vegetative state (also known as unresponsive wakefulness syndrome), minimally conscious state (MCS), and posttraumatic confusional state [30]. Each condition is defined by different levels of arousal, motor function, and purposeful behavior and is associated with its own natural history, prognosis, and management recommendations [31, 32]. DoC programs typically offer interdisciplinary care teams providing specialized physical, occupational, and speech therapy; detailed behavioral evaluations to detect subtle evidence of consciousness; communication augmentation equipment; rigorous stimulant medication trials; and training and education to help surrogates and family members provide care. Patient responses to these rehabilitative techniques help clinicians at DoC programs (e.g., neurologists, physiatrists, neuropsychologists) establish clearer estimates of a patient’s level of consciousness and their neurologic prognosis. For patients with a limited recovery trajectory and for whom prognosis remains poor, these clinicians may facilitate discussions about D-WLST.

There are principles that broadly inform the prognosis from DoC. Among DoC, higher degrees of consciousness in the acute care setting predict greater functional recovery in the future [31, 33]. Different etiologies of SABI carry different prognoses; for example, as discussed, rates of consciousness recovery and functional independence are higher following TBI than HIBI [19, 34]. Longer durations of DoC are associated with a lower likelihood of functional recovery [35]. However, in a prospective study of 484 patients with moderate to severe TBI, significant recovery, as reflected by improvements in consciousness level, disability rating scale, and/or functional independence, was possible through 1 year following injury [36]. A similar study in patients among the Traumatic Brain Injury Model Systems cohort found significant improvements in functional activities even 5–10 years post injury [17]. These data caution against the presumption of a pessimistic prognosis in the acute care setting. However, data specific to outcomes from DoC programs are sparse, and further work is necessary to determine whether and how D-WLST decisions are influenced by such programs in IRFs and LTACHs.

The phenomenon of covert consciousness, or cognitive motor dissociation (CMD), may complicate decisions about D-WLST in DoC. CMD describes a scenario in which patients without behavioral indications of awareness nonetheless demonstrate willfull modulation of brain activity to commands through functional magnetic resonance imaging or electroencephalography. An estimated 15–25% of patients with DoC may demonstrate CMD [37, 38], which is associated with improved outcomes at 3, 6, and 12 months [38, 39]. Because of its implications for current levels of awareness and future prognosis, surrogates may be interested to know a patient’s CMD status when debating whether to pursue D-WLST. However, these techniques are not yet widely available. Broader implementation of CMD detection protocols, particularly in the post-acute-care setting, may help facilitate decisions about D-WLST [40].

### Considering D-WLST

A subset of patients discharged from the hospital with DoC will not make a substantial neurologic recovery. As time goes on, a limited and plateauing recovery trajectory may predict a grim long-term prognosis with increasing likelihood [2, 35]. Depending on each patient’s wishes and values, some recoveries and prognoses will not be considered compatible with an acceptable quality of life. In these cases, patients, surrogates, and/or clinicians may consider D-WLST. Here we discuss relevant practical and theoretical factors for clinicians to consider.

### Timing Considerations

One of the most challenging considerations of D-WLST is when it should be pursued. That is, if a patient has not recovered to a state they would find acceptable, how long should surrogates and clinicians wait before concluding that a personally meaningful recovery is unlikely and thus that D-WLST is appropriate? Framed another way, when acute care providers establish the expectation of a time-limited trial (TLT) of recovery, how long should they recommend the trial to last?

In a TLT, surrogates and clinicians jointly establish specific clinical and/or functional goals, typically based on the patient’s values and wishes, to be met during a specific time frame. During that time period, recovery is supported and optimized. At the conclusion of that time period, goal attainment and the goals themselves are reassessed. If, at the conclusion of the TLT, the patient’s current neurologic status and trajectory are no longer felt to be compatible with an acceptable quality of life, D-WLST may be considered. TLTs, when communicated effectively, facilitate shared decision-making, provide families with time and space for emotional processing, and allow physicians to navigate the uncertainty of prognostication by gathering additional data in a structured manner. TLTs have been used in many intensive care settings and have the potential to reduce ICU length of stay, reduce the number of invasive procedures, and improve the frequency and quality of family meetings [41]. The utility of TLTs may be mitigated by rotating clinicians and unexpected changes in clinical course [42, 43] as well as by poor communication between clinicians and surrogates [44]. TLTs may be further complicated by patient transfers between facilities. Despite its challenges, when communicated effectively between clinicians, surrogates, and facilities, TLTs offer a standardized approach to considerations of D-WLST so that expectations for goals and a timeline are clearly established. Although the goals are typically dictated by the patient’s values, clinicians play an important role in recommending a timeline for TLTs and for reconsidering D-WLST. However, for many clinicians, appropriate timing remains uncertain.

A study of patients with moderate to severe TBI cautioned against providing definitive prognoses soon after the injury; among patients in a vegetative state at 2 weeks post injury, all recovered consciousness and 25% regained orientation by 12 months, assuming survival [36]. However, even longer periods of observation may provide additional certainty, leading guidelines to suggest a threshold of at least 28 days post injury before a poor prognosis can be more confidently concluded [7]. Given that ongoing neurologic recovery after SABI may continue for years after injury [17, 19, 36], some might argue for even more conservative (i.e., delayed) timing thresholds [34].

There are principles that may help guide this timing consideration. First, the natural history of neurologic recovery varies depending on the etiology of the injury. Nontraumatic causes of SABI, such as ischemia, hemorrhage, and HIBI, are associated with worse outcomes [7, 45], as reflected by lower survival rates, lower rates of consciousness recovery [31, 46, 47], and less robust recovery of motor and communication function [47]. Although clinical guidelines discourage the presumption of permanence (as in the now antiquated “permanent vegetative state” [7]), the relative rarity of delayed recoveries in nontraumatic etiologies has led some to define “late recovery” in these conditions as recovery beyond 3 months, in contrast to the 12-month “late recovery” threshold in TBI [34]. Thus, clinicians may feel that longer TLTs are appropriate in traumatic etiologies of DoC.

Second, patients in an MCS have better prospects of recovery than those in a vegetative state [31, 46, 48]. Thus, based on similar logic, clinicians may favor longer TLTs for patients in an MCS. Third, the patient’s personal goals may help dictate the duration of a TLT. For patients with more ambitious goals (e.g., recovery of functional independence), as compared to those with more modest goals (e.g., recovery to MCS), the feasibility of goal achievement may become clearer at earlier stages of recovery, and thus shorter TLTs may be appropriate. Fourth, tolerance of recovery duration may influence timing; for patients believed to have a lower tolerance for a protacted recovery, a shorter TLT may be appropriate. Ultimately, the timing of D-WLST is a challenging decision that must be individualized to each patient, depending on their wishes, tolerance for a protracted recovery, and details of their brain injury and recovery trajectory [2] (Table 2).

**Table 2:** Road map for approaching D-WLST

Setting	Consideration	Recommendation
Acute care setting	Which patients may be appropriate for deferring decisions about WLST until after the hospitalization?	Patients with a hopeful or uncertain prognosis Surrogates who require more time before making decision about WLST
	How may the option of D-WLST be discussed with surrogates prior to hospital discharge?	Establish patient goals Establish timeline for revisiting WLST (consider time-limited trial) Discuss logistics and psychological challenges of D-WLST Discuss possibility of regret paradox
	How may post-acute-care facilities be selected and discussed with surrogates?	Facilities typically determined by patient eligibility, bed availability, among other factors Inform family of the selected facility’s resources, goals, and timeline to establish realistic expectations Consider establishing a clinical provider(s) as a point of continuity for family and facilities to revisit discussions of prognosis and WLST Communicate plans to post-acute-care providers
Post-acute-/chronic-care setting	How may providers communicate with surrogates after hospital discharge?	Regular discussions with surrogates about patient trajectory and goals When available, interdisciplinary input (e.g., from physiatry, neurology, palliative care, social work) to discuss recovery trajectory
	When may providers consider D-WLST?	Once a recovery trajectory reveals a poor long-term prognosis (ideally following 28 days post injury) The duration, complications, or discomforts of recovery are deemed unacceptable by patients or surrogates
	How may D-WLST be implemented when deemed appropriate?	Discontinuation of life-sustaining treatments Consideration of discontinuation of artificial nutrition and hydration Defer medical escalation (e.g., rehospitalization) Liberal administration of comfort-focused interventions (e.g., analgesics, anxiolytics)

*D-WLST* delayed withdrawal of life-sustaining treatment

### Implementation Considerations

By the time D-WLST is considered, some patients will have undergone surgery for tracheostomy and gastrostomy tubes, which enable long-term ventilation and nutrition. Because most patients have medically stabilized, WLST does not typically result in immediate death. Therefore, D-WLST may be less straightforward in the post-acute-care setting than WLST during the acute care hospitalization. So what does D-WLST actually entail for patients with DoC?

D-WLST typically includes at least three components. First, any medical interventions aimed solely at the prolongation of life are often discontinued. Medications that prevent distressing symptoms (e.g., antiseizure medications) or provide comfort (e.g., analgesics or anxiolytics) are generally continued. The withdrawal of mechanical ventilatory support is often pursued, if applicable. Second, a decision is often made to not escalate care. For example, if a patient develops pneumonia, they will not be rehospitalized or given antibiotics. Third, artificial nutrition and hydration (ANH) may be withheld. Doing so can be controversial. It is legally permissible to withhold ANH; in the 1990 US Supreme Court Case *Cruzan v. Director, Missouri Department of Health*, the Court defined ANH as a medical treatment capable of being withheld when there is “clear and convincing evidence” that the patient’s wishes indicate doing so [49], and other jurisdictions [50, 51] and organizations [49] have drawn similar conclusions. Withholding ANH often facilitates the dying process (which otherwise may be more protracted) and avoids complications associated with ANH (e.g., aspiration and hypervolemia). However, ANH is sometimes viewed by family members, religious communities, and/or health care providers as emotionally comforting or palliative for even unconscious patients, and withholding it may be associated with increased feelings of guilt and shame in both family and staff [52]. Ultimately, D-WLST may consist of any combination of these three components, as appropriate for each patient, family, and institution. Of note, in contrast to WLST in the acute care setting, where surrogates decline invasive measures, such as tracheostomy and gastrostomy tube placement, D-WLST may be more psychologically challenging for surrogates because it often requires withholding interventions that are easily accessible and painless to deliver, such as medications or ANH.

### Psychosocial Considerations and the Regret Paradox

Despite limited goal attainment, it is common for surrogates to elect to continue LST [23]. Literature on patient and family attitudes toward the continuation of LST after SABI indicates that even when patients do not recover to their preestablished goals, families often do not pursue D-WLST and do not regret having continued LST [53, 54].

These observations establish an apparent “regret paradox,” that is, when surrogates decide to continue LST to meet the patient’s goals but those goals are not ultimately attained, surrogates often do not regret the decision to have continued LST and do not pursue D-WLST. There are several possible explanations for this paradox. First, after patients and surrogates discover what neurologic disability actually entails and that an acceptable quality of life may be possible despite it, their goals may change to accommodate the patient’s current state. A related phenomenon, termed the “disability paradox,” refers to the discrepancy between the poor quality of life that nondisabled individuals presume disability entails and the high quality of life often reported by those with disability [55–57]. Second, cognitive dissonance may play a role, that is, some patients and surrogates may reconcile protracted, hard-fought rehabilitation with ultimately severe disability by concluding that such disability must have been worthwhile. Third, patients and surrogates may have mentally committed to the difficult road of recovery, and it may be emotionally difficult for them to change course, akin to a sunk-cost fallacy. Fourth, the challenges of D-WLST, both practically and psychologically, may be so overwhelming to surrogates that they prefer to maintain the patient’s disabled state. Fifth, some surrogates may feel that due to psychological, cultural, or religious reasons, WLST was never a viable option in the first place. And lastly, it is possible that the data suggestive of a regret paradox are the result of a selection and survivorship bias, as patients who survive and families who opt to defer WLST may be enriched for individuals who more heavily favor LST and/or are less strongly tied to the initially established goals. It remains uncertain which of these factors, none of which are mutually exclusive, contribute most strongly to this observed regret paradox. More data will be necessary to elaborate on this phenomenon and to determine how patient and surrogates reflect on their decision-making during and after the consideration of D-WLST. Nevertheless, it is an important phenomenon to recognize and consider when counseling patients and surrogates about D-WLST, as it may be relevant for surrogates to know that perceptions of quality of life and goals may evolve over time (Table 2).

### Social Infrastructural Considerations

We acknowledge that the data and facilities reviewed in this article largely apply to the United States health care system. Important differences, such as the structure of and criteria for acceptance at rehabilitation facilities; the epidemiology of SABI, DoC, and other premorbid medical conditions; the delineation of roles between medical providers; and public, legal, and religious discourse surrounding end-of-life care, would impact how D-WLST is managed within different health care systems [58–60]. Additionally, insurance and payer systems vary between countries and might affect a patient’s approval for and duration of stay at post-acute-care facilities. And the family or surrogate’s ability to financially support prolonged medical care, whether in the acute care hospital, at a post-acute-care facility, or at home, is often an important factor in end-of-life decision-making. Finally, demographic factors, such as race, religion, socioeconomic status, and medial literacy, shape patient access to post-acute care [61], approach to end-of-life decisions, and perception and trust of health care providers [62–64]. Training clinicians to recognize fault points in equitable care and to provide culturally competent care during D-WLST decision-making is an essential aspect of effective communication and shared decision-making.

## Discussion

Acute care providers may reasonably consider delaying WLST to the post-acute-care setting for appropriate patient populations, but for many acute care providers, the practical and theoretical considerations of D-WLST are unknown. The reviewed literature and guidelines highlight concepts for acute care providers to recognize when counseling surrogates and families about the option of D-WLST (Table 2). First, the chances of an adequate recovery and thus the need to consider D-WLST vary widely between patients. The type of injury, the severity of the injury, the medical comorbidities, the certainty of prognosis, the discharge disposition, the patient’s goals and values, and the surrogate’s need for time to process the situation are among many factors that influence the relevance of D-WLST discussions.

Second, clinicians may consider establishing a timeline and protocol through which surrogates and/or patients can discuss and pursue D-WLST if appropriate, particularly if their post-acute-care facility does not routinely offer the clinical support for doing so. Ideally, such support would include interdisciplinary input (e.g., from palliative care, physiatry, neurology, and social work), but the feasibility of this approach will depend on the institution and the capacity to communicate between facilities [41–44]. TLTs may be an effective tool for establishing that timeline, facilitating joint decision-making between clinicians and families, and standardizing the transition from the acute care to post-acute-care setting. However, as discussed, TLTs are not without their own challenges. An increasing number of facilities have begun to offer longitudinal models of care for SABI [65], including neurorecovery clinics, where neurointensivists continue to provide longitudinal care to their patients in the outpatient setting [66, 67]. These models improve continuity and support to patients and families, and may facilitate TLTs and consideration of D-WLST when appropriate.

Third, clinicians should be mindful of the “regret paradox” and consider that the failure to achieve goals initially established by surrogates in the acute care setting may not lead to D-WLST after discharge. Co-survivorship is a complex role for surrogates to assume, with many psychological, emotional, financial, and practical components. All together, consideration of these concepts may help promote informed and shared decision-making between clinicians and surrogates. Clear communication about the patient’s clinical status and trajectory, clarity about what continued treatment and WLST entail, and agreement on important clinical milestones in the acute care and post-acute-care setting all help ensure that patients and surrogates receive the care and establish the expectations that they need to navigate the recovery process.

It is important to emphasize that not all SABI result in DoC or require consideration of WLST. Moreover, the recovery of consciousness may not be the only factor that determines whether LST is continued or withdrawn. Other relevant factors may include the recovery of other neurologic functions; the duration and type of medical treatments required to promote recovery; and other cultural, religious, and personal values that may influence goals of care. Ultimately, though the principles discussed provide a general road map for consideration of WLST, these decisions must always be tailored to the specific circumstances and values of each patient. Furthermore, for the purposes of this article, we have defined D-WLST as WLST that occurs outside the acute care hospitalization to highlight challenges in anticipating and transitioning care to the post-acute-care setting. However, certain clinical factors, such as medical readiness for discharge, or nonclinical factors, such as insurance status or regional health care infrastructural differences, may preclude select patients from transitioning to post-acute-care facilities. In these cases, many of these considerations may be similar but would apply within the acute care hospitalization as opposed to between facilities.

Future research may help clinicians and surrogates approach D-WLST discussions and decision-making. Much of the data from post-acute-care facilities are not specific to DoC resulting from SABI, which carry unique challenges. Therefore, it will be useful to further study patient outcomes and predictors of these outcomes for patients discharged with SABI and DoC to different post-acute-care facilities. Such studies would equip acute care providers and surrogates with more accurate expectations about D-WLST in the post-acute-care period. Additionally, determining how to optimally discuss WLST and D-WLST with surrogates to ensure the best functional and emotional outcomes remains a topic of investigation, with options including decision aids [68], designated family advocates [53], or road maps of post-acute-care trajectories. In all of these paths of future research, outcomes should not only include patient morbidity and mortality but should also reflect family and surrogate experiences and satisfaction with provider communication.

As clinicians begin waiting for longer periods after SABI before definitively concluding a poor neurologic prognosis, D-WLST has become an increasingly pertinent consideration. By highlighting data relevant to this decision, we hope to equip acute care providers with the necessary information to counsel families and surrogates. Clinical innovation and further research will be important to facilitate effective discussions and guide patients and families through the challenges of D-WLST decisions.
